# Bacterial Communities Associated with Four Cyanobacterial Genera Display Structural and Functional Differences: Evidence from an Experimental Approach

**DOI:** 10.3389/fmicb.2016.01662

**Published:** 2016-10-24

**Authors:** Lin Zhu, Anouk Zancarini, Imen Louati, Silvia De Cesare, Charlotte Duval, Kevin Tambosco, Cécile Bernard, Didier Debroas, Lirong Song, Julie Leloup, Jean-François Humbert

**Affiliations:** ^1^State Key Laboratory of Freshwater Ecology and Biotechnology, Institute of Hydrobiology, Chinese Academy of SciencesWuhan, China; ^2^UPMC-INRA, iEES-Paris UMR 7618Paris, France; ^3^CNRS, MCAM, Muséum National d’Histoire Naturelle, UMR 7245, Sorbonne UniversitésParis, France; ^4^Laboratoire Microorganismes: Génome et Environnement, Université de Clermont-FerrandClermont-Ferrand, France

**Keywords:** bacterial communities, cyanobacteria, cultures, natural samples, functional and structural diversity, pyrosequencing, EcoPlates

## Abstract

To overcome the limitations associated with studying the interactions between bacterial communities (BCs) and cyanobacteria in natural environments, we compared the structural and functional diversities of the BCs associated with 15 non-axenic cyanobacterial strains in culture and two natural BCs sampled during cyanobacterial blooms. No significant differences in richness and diversity were found between the natural and cultivated BCs, although some of the cyanobacterial strains had been isolated 11 years earlier. Moreover, these BCs shared some similar characteristics, such as a very low abundance of *Actinobacteria*, but they display significant differences at the operational taxonomic unit (OTU) level. Overall, our findings suggest that BCs associated with cyanobacteria in culture are good models to better understand the interactions between heterotrophic bacteria and cyanobacteria. Additionally, BCs associated with heterocystous cyanobacterial strains cultivated in Z8X culture medium without nitrate (*Aphanizomenon*–*Dolichospermum*) demonstrated significant differences compared to BCs associated with non-heterocystous strains cultivated in Z8 culture medium (*Planktothrix*–*Microcystis*) in terms of their composition and their ability to utilize different carbon sources, suggesting the potential influence of cyanobacterial metabolism and/or culture media on associated BCs. Finally, half of the dominant OTUs in these BCs were specifically associated with cyanobacteria or other phytoplankton, whereas the remaining OTUs were generally associated with ecosystems containing high organic matter content, such as sludge or intestines.

## Introduction

In the last 10 years, a growing number of published studies have investigated the bacterial communities (BCs) associated with phytoplankton. In marine ecosystems, a limited number of heterotrophic bacterial lineages displaying distinct metabolic strategies dominate these BCs (see review of Buchan et al., 2014). In freshwater ecosystems, the majority of studies have focused on cyanobacterial blooms due to their potential impact on human health (e.g., Dodds et al., 2009). For example, it has been reported that epiphytic bacteria and archaea are directly associated with cyanobacteria (e.g., Stevenson and Waterbury, 2006; Grossart et al., 2011; Woodhouse et al., 2013), and that several bacterial species degrade cyanotoxins to use them as carbon or nitrogen sources (e.g., Maruyama et al., 2004; Zhu et al., 2014). Several publications have also evaluated the impact of cyanobacterial blooms on the structure and composition of BCs within the same ecosystems (e.g., Eiler and Bertilsson, 2004; Berg et al., 2009; Louati et al., 2015; Woodhouse et al., 2016). *Microcystis* sp., a toxic bloom-forming cyanobacteria, has been a specific focus given its high prevalence in many ecosystems around the world as well as its colonial organization, which offers a potential niche and physical support for direct interactions with heterotrophic prokaryotes (e.g., Shen et al., 2011; Li et al., 2012; Shi et al., 2012; Shao et al., 2014).

Most studies are performed on natural samples and have produced interesting findings. For example, BCs associated with cyanobacteria are distinct from those that are free-living, implying direct interactions between cyanobacteria and bacteria (Cai et al., 2013; Parveen et al., 2013). However, the impact of numerous environmental factors and processes on BCs in natural environments (see Dziallas and Grossart, 2011) makes it difficult to separate the influence of cyanobacteria from that of the environment. To overcome these constraints, we compared the functional and structural diversities of BCs associated with 15 non-axenic cyanobacterial strains belonging to four genera: *Microcystis, Planktothrix, Aphanizomenon*, and *Dolichospermum*.

We addressed two primary goals to our study. The first goal was to estimate the potential utility of cultivated cyanobacterial strains to study the relationships between cyanobacteria and bacteria by comparing (i) the richness and diversity within the BCs associated with cyanobacteria in culture to estimate any decrease in these two parameters in BCs associated with cyanobacterial strains with respect to the isolation date of these strains, and (ii) the structure and composition of BCs associated with two strains (one *Microcystis* and one *Dolichospermum*) in culture compared to natural BCs sampled from the pond from which these two strains were isolated to determine whether culture conditions had a deep impact on the BCs associated with cyanobacteria. The second goal of this study was to identify the major factors influencing the structural and functional diversity of BCs associated with the 15 cultured cyanobacterial strains based on different strain characteristics such as their taxonomic affiliation, culture time, N_2_-fixing capacity (heterocystous versus non-heterocystous strains) and media composition used for the culture of these cyanobacteria.

## Materials and Methods

### Experimental Design

The 15 selected non-axenic cultures were obtained from the Paris Museum Collection (PMC) of cyanobacteria. A description of the different genera and geographical origins is provided in **Table 1**. All cyanobacterial strains were cultured under the same conditions as the PMC collection of cyanobacteria (25°C and illumination of 30 μmol quanta/m^2^/s with a photoperiod of 16/8 h of day/night). Cultures were maintained in 250-mL Erlenmeyer flasks containing 100 mL of Z8 medium (Kotai, 1972) for *Microcystis* and *Planktothrix* strains and Z8X medium (without nitrate but with ammonia in trace element solution) for N_2_-fixing *Dolichospermum* and *Aphanizomenon*. We chose to cultivate the N_2_-fixing (N_2_+) and non-N_2_-fixing (N_2_-) cyanobacterial strains in two different media to maintain continuity, as they had been cultured in these media since their isolation, and we did not want to disturb the cyanobacteria and their associated BCs by changing the culture conditions. All experiments were performed in triplicate batch cultures. The cultures were sampled after 27 days, during the logarithmic growth phase.

**Table 1:** Characteristics of the cyanobacterial strains and environmental samples used in this study.

Strain code	Strain number	Genus/species	Origin	Isolation date PMC	Toxin production	Culture medium	OD_665_ (nm)
A1	PMC 635.10	*Aphanizomenon gracile*	LCM	2010	STX +	Z8X	0.17
A2	PMC 644.10	*Aphanizomenon gracile*	LCM	2010	STX -	Z8X	0.18
D1	PMC 207.03	*Dolichospermum flos-aquae*	Senegal	2003	Anatoxin-a +	Z8X	0.09
D2	PMC 200.03	*Dolichospermum planctonicum*	Senegal	2003	STX -; CYN -; MC -; Anatoxin-a -	Z8X	0.08
D3	PMC 624.10	*Dolichospermum planctonicum*	LCM	2010	STX -	Z8X	0.03
M1	PMC 155.02	*Microcystis aeruginosa*	Senegal	2002	MC -	Z8	0.08
M2	PMC 250.05	*Microcystis aeruginosa*	Burkina Faso	2005	MC -	Z8	0.09
M3	PMC 728.11	*Microcystis*	Valence, France	2011	MC +	Z8	0.09
M4	PMC 568.08	*Microcystis*	LCM	2008	Und.	Z8	0.04
M5	PMC 828.12	*Microcystis*	LCM	2012	MC +	Z8	0.06
P1	PMC 75.02	*Planktothrix agardhii*	NBV	2002	MC +	Z8	0.18
P2	PMC 101.02	*Planktothrix agardhii*	NBV	2002	STX -; CYN -; MC -; Anatoxin-a -	Z8	0.05
P3	PMC 427.08	*Planktothrix agardhii*	Enghien, France	2008	MC -	Z8	0.14
P4	PMC 255.05	*Planktothrix agardhii*	Burkina Faso	2005	STX -; CYN -; MC -; Anatoxin-a -	Z8	0.08
P5	PMC 686.10	*Planktothrix agardhii*	Tunisia	2010	MC -	Z8	0.06
Env_D	Environmental bloom	*Dolichospermum* sp.	LCM	2012	Undetermined	-	-
Env_M	Environmental bloom	*Microcystis* sp.	LCM	2012	MC +	-	-

*PMC, Paris Museum Collection; LCM, Lake of Champs-sur-Marne, France; NBV, Nautical Base of Viry Châtillon, France; MC, microcystin; STX, saxitoxin; CYN, cylindrospermopsin; Und., undetermined; OD_*665*_, mean of the three culture replicate optical density measurements of the cyanobacterial cultures at 665 nm minus those at 750 nm on the sampling day.*

For each culture, a double filtration was performed. One hundred milliliters of each culture sample was first filtered through a 1.2-μm pore-size filter membrane (Isopore Membrane Filters, Millipore) to remove cyanobacterial cells; then, 20 mL of this filtrate was utilized for a Biolog EcoPlate experiment. Finally, 80 mL of filtrate was aseptically filtered through a 0.2-μm pore-size filter (Track-Etched Membranes, Whatman^®^Nuclepore^TM^). These filters were stored at -20°C for further molecular analyses. This removal step by filtration was required to access to the major BCs associated to cyanobacteria in cultures, but in return, bacteria attached to organic particles or to cyanobacteria might have also been removed. However, considering that these attached bacteria have a free-living phase and that the carbon sources available for heterotrophic bacteria were only provided by cyanobacteria, we have considered that the sampled BCs were directly influenced by cyanobacteria in our cultures.

Environmental cyanobacteria were collected from a recreational lake located near the city of Champs-sur-Marne (France, 48°51′47.0 N, 02°35′53.9 E) in 2012. Two cyanobacterial genera bloomed during the summer season, *Dolichospermum* in July and *Microcystis* in September, and they were sampled as previously described (Pascault et al., 2015). The cultivated *Microcystis* strain M5 (**Table 1**) used in this study was also isolated at the same time, while the *Dolichospermum* strain D3 (**Table 1**) was isolated from this lake 2 years prior. Natural bloom samples were counted by microscope, and 95% of cells belonged to *Dolichospermum* during the bloom of this genus, while the *Microcystis* bloom sample consisted of 84% *Microcystis* and 15% *Pseudoanabaena mucicola*.

### Carbon Source Utilization

The functional diversity of BCs associated with cyanobacterial strains in culture was assessed by implementing a Biolog EcoPlate approach (EcoPlates; Biolog, Hayward, CA, USA). The Biolog EcoPlate experiment used 96-well microtiter plates containing a total of 31 carbon sources and a blank containing only water, all in triplicate. In addition to the carbon source, each well-contained the redox agent tetrazolium violet as an indicator of substrate oxidation. Positive wells developed a purple color that was quantified spectrophotometrically. Each well was inoculated with 150 μL of sample, and the plates were then incubated at 25°C for 1 week. During the incubation, the absorbance of the plates was measured every day at a wavelength of 590 nm using a spectrophotometric microplate reader (Molecular Devices, USA).

The optical density in each well was averaged among technical replicates and among culture triplicates. The carbon metabolic activity of the BCs was analyzed for all 31 carbon sources using principal component analysis (PCA). For each BC associated with the different cyanobacterial strains, microbial activity was expressed as the average well-color development (AWCD), determined as follows: AWCD = ΣOD_i_/31, where OD_i_ is the mean optical density value from each well. Carbon metabolic richness was calculated as the number of oxidized carbon substrates using an OD of 0.25 as the threshold for a positive response (Garland, 1996). The nitrogen use index (NUI) was calculated and expressed as the ratio of total substrate utilization to substrates containing nitrogen (Sala et al., 2006).

### Molecular Approaches

The structural diversity of BCs associated with cyanobacterial strains in culture and with natural blooms of cyanobacteria was assessed using a 16S rRNA gene fragment pyrosequencing approach. The DNA extraction procedure involved mechanical and chemical extraction and was adapted from the procedure described by Massana et al. (1997). Each 0.2-μm filter was resuspended in 1.1 mL of lysis buffer (40 mM EDTA, 50 mM Tris-HCl, 0.75 M sucrose) and crushed using Lysing Matrix E tubes in a Fast Prep (MP Biomedical, Fast Prep Instrument^®^-24). In addition, enzymatic lysis was performed by adding lysozyme (0.5 mg/mL). After 45 min at 37°C, deproteinization was carried out by bringing the mixture to a final concentration of 10% sodium dodecyl sulfate (SDS) and adding proteinase K (0.2 mg/mL, Thermo Scientific) for 1.5 h at 55°C. Then, samples were centrifuged, and the supernatants were collected and purified with phenol-chloroform-isoamyl alcohol followed by chloroform. After precipitating with a 0.1 sample volume of sodium acetate and a 0.6 sample volume of isopropanol, the nucleic acids were washed with 80% ethanol and re-suspended in 100 μL of ultrapure water. The samples were stored at -20°C.

A region of the 16S rRNA gene including the variable region V4–V5 was selected for tag pyrosequencing. This region was amplified using the bacterial forward primer 563F (Claesson et al., 2010), which also includes the Roche 454 pyrosequencing adapter FLX A and a unique 10-bp barcode, and the bacterial reverse primer 907rm (Schauer et al., 2003), which includes the Roche 454 pyrosequencing adapter FLX B. Polymerase chain reaction (PCR) was performed under the following conditions: 98°C for 2 min; 30 cycles of 98°C for 10 s, 52°C for 30 s and 72°C for 1 min; 72°C for 10 min. Each DNA sample was amplified in two replicate PCR reactions, which were then pooled. These PCR products were then purified using the MinElute Gel Extraction Kit (Qiagen, Venlo, Pays-Bas). The amount of DNA in each sample was quantified using the Qubit dsDNA HS assay (Invitrogen, Carlsbad, CA, USA). Finally, the PCR products were combined in equimolar amounts and sequenced using a Genome Sequencer GS FLX Titanium 454 of GATC Biotech (Roche Company, Branford, CT, USA).

### Bioinformatics Analysis

The 454 pyrosequencing of the 16S rRNA gene produced 514,905 raw sequences. All sequences were cleaned by applying PANGEA trimming (Giongo et al., 2010) with a quality threshold (>23) and a minimum sequence length of 270 bp and by removing sequences with errors in the forward primer. The remaining sequences were clustered using USEARCH (Edgar, 2010) at a 97% similarity threshold and were then grouped according to different taxonomic levels using the SILVA base (Pruesse et al., 2007). The LCA (Lowest Common Ancestor) assignment method was employed. The process was automated by PANAM pipeline^1^, which constructs phylogenetic trees for taxonomic annotation (Taib et al., 2013). After the removal of low-quality sequences, a range of 2,127 to 17,266 sequences was obtained for each BC associated with different cyanobacterial strain triplicates. After removing the cyanobacterial sequences (representing approximately 25% (± 22%) of the total bacterial sequences among the different samples) and singletons (every sequence found only once in the entire data set), 1,005 to 16,383 sequences were retrieved per sample. For further analyses, sequence data sets were normalized to 1,004 sequences. Within the 50,200 sequences analyzed (1,004 sequences per sample), a total of 1,125 operational taxonomic units (OTUs) were obtained from cyanobacteria-associated BCs in culture, and 1,458 OTUs were obtained from combined analyses of cyanobacteria-associated BCs in culture and those from the two natural bloom samples. OTU thresholds were defined by an abundance of reads >1% within a pooled sample corresponding to an abundant OTU, whereas a proportion <0.01% represented a rare OTU, as described in Pedrós-Alió (2006) and Galand et al. (2009).

### Statistical Analysis

All statistical analyses were performed using the statistical software package R 2.15.1 (R Development Core Team, Vienna, Austria). Only differences at *p* < 0.05 were considered significant.

To test the potential effects of the different characteristics of the cyanobacterial strains (strain, genus, sampling date, putative toxicity, N_2_ fixation, and culture medium) on the different variables studied (AWCD, carbon metabolic richness, NUI, the three first PCA axes of the two PCAs based on EcoPlate and 454 pyrosequencing data), we first chose the best model according to the Akaike Information Criterion (AIC) in a Stepwise Algorithm using the “step” function in R, and we then calculated type II analysis-of-variance (ANOVA) tables. We took into account certain factors that were not independent, such as the cyanobacterial strain and genus or N_2_ fixation and culture medium. Then, differences in carbon substrate use, the NUI, the AWCD and the carbon metabolic richness according to the cyanobacterial genera were tested by ANOVA and Tukey’s tests.

Principal component analysis and co-inertia analyses were performed using Ade4TkGUI software (Thioulouse and Dray, 2007). A Hellinger transformation had previously been carried out on the 454 pyrosequencing data using the vegan package (Dixon, 2003). Permutational multivariate ANOVA (PERMANOVA) and Mantel tests were performed using the vegan package to test for differences between BCs associated with N_2_+ cyanobacteria and those associated with N_2_- cyanobacteria. Diversity indices were calculated using the vegan package with the normalized data set (Dixon, 2003).

The effects of N_2_ fixation/culture medium on the distribution of the major bacterial orders and classes and on the dominant bacterial OTUs (sequence numbers > 1% of the total sequences) associated with the different cyanobacterial strains were analyzed by performing ANOVA followed by Tukey’s tests.

A network approach was implemented to visualize OTU distribution among the different cyanobacterial strains. The network was constructed based on the presence/absence of data for each OTU in all cyanobacterial cultures after pooling the replicates. Cyanobacterial strains were used as source nodes, and OTUs were used as target nodes, with edges (that is, lines connecting nodes) corresponding to positive associations between OTUs and cyanobacterial strains. Then, the network was visualized using the edge-weighted spring-embedded layout algorithm in Cytoscape Software (Shannon et al., 2003).

## Results

### Richness and Diversity in BC Associated with Cyanobacteria in Culture and in Natural Blooms of Cyanobacteria

As shown in **Table 2**, the richness and diversity values of BCs associated with the two cultured strains *Dolichospermum* D3 and *Microcystis* M5 were substantially lower than those of BCs associated with natural blooms of these two genera in the lake from which these two strains were isolated. However, when all values obtained for the 15 strains in culture and for the two natural blooms were compared, no significant differences in the diversity indices of these BC types were noted (Kruskal–Wallis tests; *p* = 0.06, 0.06, and 0.19 for richness, Chao1 and Shannon, respectively; **Table 2**). Furthermore, there were no significant differences in the richness and diversity of BCs associated with cyanobacterial strains isolated before or after 2008 (Kruskal–Wallis tests; *p* = 0.94, 0.80, and 0.54 for richness, Chao1 and Shannon, respectively).

**Table 2:** Richness, Chao1 and diversity values estimated for the bacterial communities associated with cyanobacteria in culture and with the two environmental samples.

	Richness	Chao 1	Shannon
*Dolichospermum* (D3) in culture	91 ± 8	202 ± 41	3.4 ± 0.1
Environmental *Dolichospermum*	125 ± 8	219 ± 46	3.3 ± 0.6
*Microcystis* (M5) in culture	61 ± 15	112 ± 51	2.7 ± 0.5
Environmental *Microcystis*	179 ± 5	242 ± 15	4.5 ± 0.1
*Dolichospermum* in culture	85 ± 24	170 ± 63	3.1 ± 0.4
*Microcystis* in culture	50 ± 10	89 ± 17	2.3 ± 0.4
*Aphanizomenon* in culture	101 ± 31	220 ± 89	3.2 ± 0.2
*Planktothrix* in culture	73 ± 16	113 ± 25	2.8 ± 0.2

*Diversity indices were first estimated for each sample using the vegan package in R. For *Dolichospermum* (D3) in culture, *Microcystis* (M5) in culture, environmental *Dolichospermum* and environmental *Microcystis* samples, standard deviations were estimated from the three replicates. For *Dolichospermum, Microcystis, Aphanizomenon*, and *Planktothrix* in culture, we first estimated the mean values of the diversity indices for each strain based on three replicates and then estimated the standard deviation of the diversity indices for each genus using the mean values obtained for each strain.*

**FIGURE 1:**
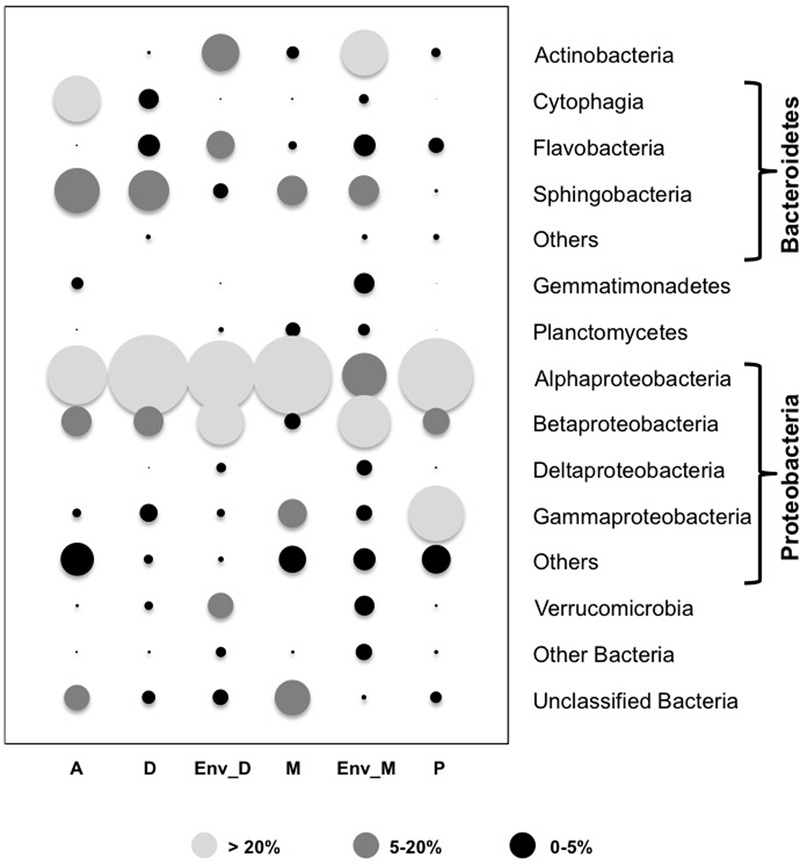
**Relative abundance of main groups found in the bacterial communities (BCs) associated with the four cyanobacterial genera in culture and with the two natural blooms of cyanobacteria after normalization to the smallest sample (*n* = 1004).** The size of the circles was estimated after averaging in a first time, the number of reads found in the three replicates for each strain and in a second time the mean number of reads found in each strain of a given genus. A: *Aphanizomenon* strains; D: *Dolichospermum* strains; M: *Microcystis* strains; P: *Planktothrix* strains; Env_D: Environmental sample of an *Dolichospermum* bloom; Env_M: Environmental sample of a *Microcystis* bloom; Others (within the *Bacteroidetes* and the *Proteobacteria* sections): all the sequences of minority classes (<1% for all the Cyanobacterial genera) and also included for the Proteobacteria section, sequences only identified as Proteobacteria using the SILVA base and the assignment LCA (Lowest Common Ancestor); Other Bacteria: all the sequences of non-dominant phyla (<1% for all the Cyanobacterial genera); Unclassified Bacteria: sequences only identified as Bacteria using the SILVA by the LCA method.

**FIGURE 2:**
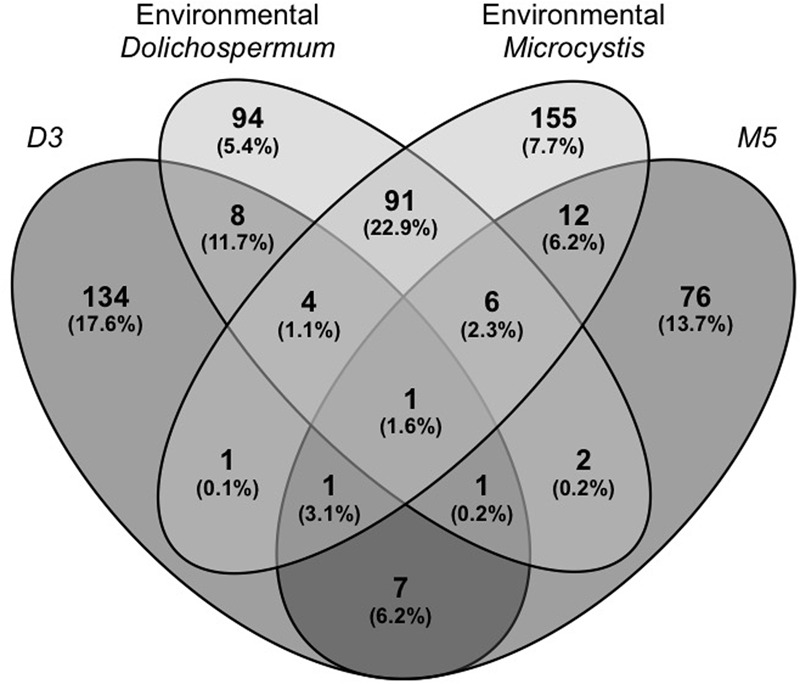
**Venn diagram showing the distribution and abundance of the bacterial OTUs found in the BCs sampled during the two natural blooms of *Microcystis* and *Dolichospermum* and in the BCs associated with the *Microcystis* (M5) and *Dolichospermum* (D3) strains in culture.** The abundances (total read percentages) of these OTUs are represented in brackets. The number of reads was first added for the three replicates, and their abundances were then calculated for the different OTU groups based on total OTUs for these four cyanobacteria, both for natural blooms and individual strains (*n* = 12,048 reads). D3: BC associated with *Dolichospermum* strain 3 (isolated from Champs/Marne); M5: BC associated with *Microcystis* strain 5 (isolated from Champs/Marne); Environmental *Dolichospermum*/*Microcystis*: BC associated with the two bloom samples of *Dolichospermum* and *Microcystis* from Champs/Marne.

Comparison of the global composition and structure of the BCs associated with cyanobacteria in culture and those associated with natural blooms of *Dolichospermum* and *Microcystis* revealed a shared dominance by *Proteobacteria*, particularly *Alphaproteobacteria*, and *Bacteroidetes* in these communities. These BCs primarily differed in terms of the relative abundances of *Actinobacteria* and *Betaproteobacteria* and, to a lesser extent, *Verrucomicrobia* (**Figure 1**); these phyla were more abundant in BCs associated with cyanobacteria in natural blooms than those in culture. At the OTU level, significant differences were found between natural and cultivated BCs, as these BCs shared only 5.6% of their OTUs (**Figure 2**). However, these shared OTUs contained more than 26.2% of all sequences; thus, the majority of these shared OTUs were likely dominant in all BCs (**Figure 2**). Interestingly, PCA performed on all data revealed the distribution of BCs in three different groups: BCs from environmental samples, BCs from N_2_+ cyanobacteria in culture and BCs from N_2_- cyanobacteria in culture (**Supplementary Figure S1**).

### Comparison of the Carbon and Nitrogen Metabolic Activities of BCs Associated with Cyanobacterial Cultures

**FIGURE 3:**
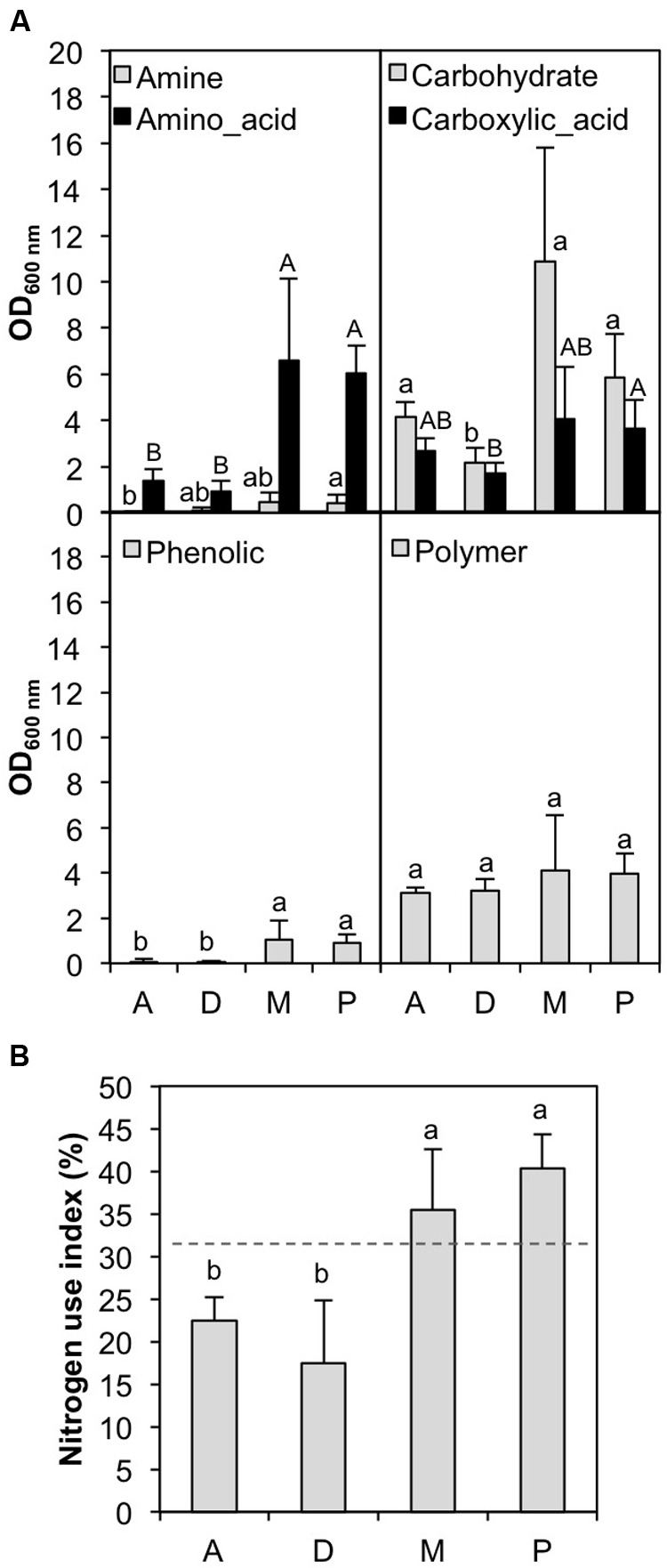
**Comparative use of **(A)** different families of carbon substrates available in the Biolog EcoPlates and **(B)** nitrogen use index by the BCs associated with cyanobacterial strains in culture.** D: *Dolichospermum* strains; A: *Aphanizomenon* strains; M: *Microcystis* strains; P: *Planktothrix* strains. Letters with different labels indicate significant differences (*p* < 0.05).

To determine whether BCs associated with different cyanobacterial strains in culture exhibited divergent catabolic profiles, we implemented a Biolog EcoPlate approach. The number of carbon substrates and the AWCD were significantly higher (ANOVA analysis: df = 1, *F*-value = 126.1, *p* = 2.4 10^-10^ and df = 1, *F*-value = 198.8, *p* = 3.5 10^-12^, respectively) for BCs associated with N_2_- cyanobacteria (*Microcystis* and *Planktothrix* strains) cultivated in Z8 medium than for those associated with N_2_+ cyanobacteria (*Dolichospermum* and *Aphanizomenon* strains) cultivated in Z8X medium. More specifically, after grouping every carbon source by chemical category, amino acids, carbohydrates and phenolic substances were highly consumed by BCs associated with N_2_- cyanobacteria (**Figure 3A**). Similarly, the NUI (Urakawa et al., 2013) was significantly higher for BCs associated with N_2_- cyanobacteria than for those associated with N_2_+ cyanobacteria (ANOVA analysis: df = 1, *F*-value = 289.5, *p* = 9.3 10^-14^) (**Figure 3B**). Overall, among the different characteristics of the cyanobacterial strains (N_2_ fixation, culture medium, date of isolation, genus and putative toxicity), only N_2_ fixation/culture medium and the interactions among cyanobacterial strain, genus and N_2_ fixation/culture medium had significant effects on carbon and nitrogen metabolic activities [ANOVA analyses using best models given by AIC analyses (∼Fixation + Fixation:Strain:Genus), df = 1 and *F*-value > 126.1 for N_2_ fixation; df = 13 and *F*-value < 18.1 for Fixation:Strain:Genus, *p* < 0.001].

**FIGURE 4:**
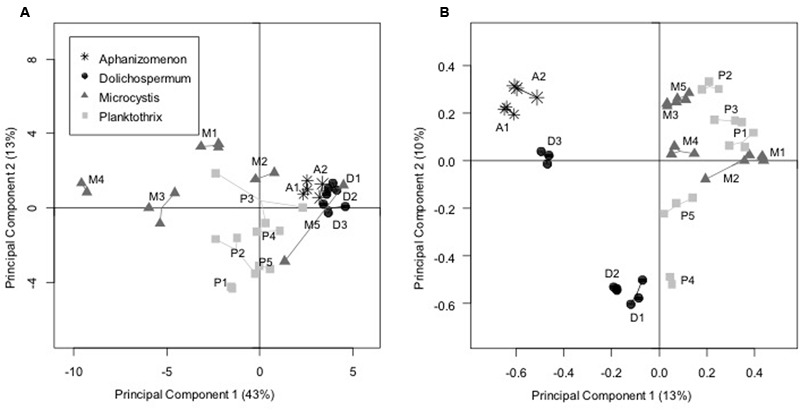
**Principal component analyses performed **(A)** on the use of the 31 carbon sources (EcoPlate approach) and (B) on the OTU composition (pyrosequencing approach) of the BCs associated with the cyanobacterial strains in culture.** For each strain, the replicates were connected together in order to facilitate the reading. A: *Aphanizomenon* strains; D: *Dolichospermum* strains; M: *Microcystis* strains; P: *Planktothrix* strains.

Finally, PCA revealed a clear distinction between the BCs associated with *Dolichospermum* and *Aphanizomenon* strains that grouped together and those associated with *Planktothrix* and *Microcystis* strains (PERMANOVA analysis, df = 1, *R*^2^ = 0.35, *p* = 0.0001; **Figure 4A**). Furthermore, the BCs associated with different *Microcystis* strains and, to a lesser extent, the *Planktothrix* strains demonstrated higher carbon source variability than those associated with the *Dolichospermum* and *Aphanizomenon* strains.

### Comparison of the Structure and Composition of BCs Associated with Cyanobacterial Cultures

Based on overall BC structure, *Proteobacteria* (particularly *Alphaproteobacteria*) and *Bacteroidetes* were the two most abundant phyla in all culture samples (**Figure 1**), while the read numbers of other phyla did not exceed 2% (0.7–2.0%). Even if the global structures of BC communities were very similar, regardless of the cyanobacterial genera, some groups were preferentially detected in only one cyanobacterial genus (**Figure 1**). For example, *Planctomycetes* were primarily found in BCs associated with *Microcystis* strains, whereas *Cytophaga* (*Bacteroidetes*) were primarily found in heterocystous cyanobacterial cultures.

**FIGURE 5:**
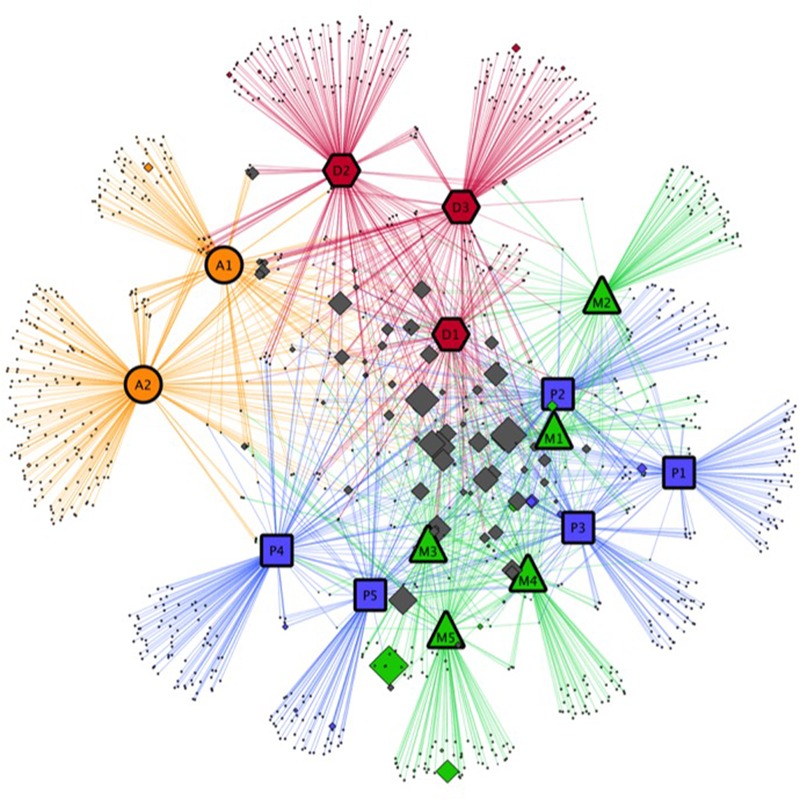
**Network analysis on the relationships between cyanobacterial strains in culture and the bacterial OTU identified in our study.** Colored symbols with large black border represent the different cyanobacterial strains: orange circles, red hexagons, green triangles and blue rectangles represent respectively *Aphanizomenon* strains (A), *Dolichospermum* strains (D), *Microcystis* strains (M), and *Planktothrix* strains (P). Diamonds represent the bacterial OTU found in associated with cyanobacterial strains. Colored diamonds are used for bacterial OTU associated with only one cyanobacterial strain (for example, green diamonds correspond to bacterial OTU only associated with one *Dolichospermum* strain). Gray diamonds represent OTU associated with several cyanobacterial strains. The size of the diamonds is proportional to the relative abundance of the OTU.

When the BCs associated with the 15 cyanobacterial strains at the OTU level were compared in our network analysis (**Figure 5**), almost all abundant OTUs were shared by several BCs (OTUs located at the center of **Figure 5**), while numerous OTUs with low abundance were generally found in only one BC (OTUs located at the periphery of the **Figure 5**).

Special attention was focused on the distribution of the 100 most abundant OTUs. Forty seven of these OTUs demonstrated a significant difference (ANOVA analysis, *p* < 0.05) in their relative abundance when comparing BCs associated with N_2_+ cyanobacteria cultivated in Z8X medium and those associated with N_2_- strains cultivated in Z8 medium. Second, BLAST analysis was performed with these 100 OTUs in the GenBank^TM^ (GB) database to determine how many sequences exhibited 98% sequence identity across more than 98% of their sequence length to our 100 OTUs. The taxonomic assignments of these OTUs obtained via BLAST analysis were congruent with those obtained from the SILVA database (see Materials and Methods). Interestingly, for approximately 50% of our OTUs, only a limited number of matches were found in GB (less than 50 sequences for each OTU), whereas for the rest of the OTUs, numerous matches were found in GB (at least 100 sequences but frequently several 100). In instances where few sequences were retrieved from GB, almost all were associated with freshwater ecosystems, and the majority of these OTUs were associated with cyanobacterial blooms (**Supplementary Table S1**). Conversely, when more than 100 sequences were retrieved from GB, these sequences originated from various ecosystems that were generally characterized by a high level of organic matter (OM), including freshwater ecosystems but also wastewater sludge, sediment, human waste and skin, and soils (**Supplementary Table S1**). In addition to their affinity for OM, we paid special attention to the potential ability of these dominant bacterial OTUs associated with cyanobacteria in culture to fix free nitrogen. Within the dominant OTUs, we identified OTUs belonging to three genera (*Rhizobium, Azospirillum*, and *Pseudomonas*) known to contain potentially N_2_-fixing species. Their abundance in each cyanobacterial genus varied widely according to the strain and replicate (from 0 to 23.5% in *Dolichospermum*, 0 to 7.2% in *Aphanizomenon*, 0 to 37.6% in *Microcystis*, and 0 to 43.3% in *Planktothrix*). *Rhizobium* and *Pseudomonas* were primarily associated with N_2_- cyanobacteria while *Azospirillum* was primarily associated with N_2_+ cyanobacteria.

Principal component analysis performed on the distribution of all bacterial OTUs in BCs associated with the 15 cyanobacterial strains (in triplicate) revealed a significant difference (PERMANOVA analysis: df = 1, *R*^2^ = 0.14, *p* = 0.0001) along the first axis of the analysis (**Figure 4B**) between BCs associated with N_2_+ cyanobacteria cultivated in Z8X medium and those associated with N_2_- cyanobacteria cultivated in Z8 medium. When testing the potential effects of certain cyanobacterial strain characteristics (N_2_ fixation, culture medium, strain, genus, sampling date, and putative toxicity), only N_2_ fixation/culture medium and the interactions among cyanobacterial strain, genus, and N_2_ fixation/culture medium had significant effects on the BC distribution along the first two PCA axes (ANOVA analyses using best models given by AIC analyses (∼Fixation + Fixation:Strain:Genus), df = 1, *R*^2^ = 0.14, *p* = 0.0001 for N_2_ fixation and df = 13, *R*^2^ = 0.74, *p* = 0.0001 for interactions among cyanobacterial strain, genus, and N_2_ fixation/culture medium). Finally, a co-inertia analysis revealed a significant correlation between the two PCA analyses performed on the 454 pyrosequencing and on the EcoPlate data (Mantel test: *R*^2^ = 0.45, *p* = 0.0001).

## Discussion

With regard to the first aim of our study, which investigated the potential utility of cultivated cyanobacterial strains to study the relationships between cyanobacteria and bacteria, our data indicate a high level of richness and diversity maintained in BCs associated with cultured cyanobacteria, even in strains isolated a long time ago. Two major processes may be involved in the conservation of BC diversity. First, changes in the quality and the quantity of OM produced by cyanobacteria during growth (Leloup et al., 2013) may cause temporal successions in BCs (Bagatini et al., 2014; Brauer et al., 2015), maintaining a large degree of richness and diversity. Second, the influence of bacteriophages on the dynamics and diversity of BCs may also sustain a high level of richness in our cultured communities (see reviews of Wommack and Colwell, 2000; Sime-Ngando, 2014).

In addition to these findings, the comparison of BC communities associated with cyanobacterial cultures and those living in freshwater ecosystems hosting cyanobacterial blooms revealed several shared similarities at the phylum level, specifically a low proportion of *Actinobacteria* and a relatively high abundance of *Gammaproteobacteria* in addition to the dominance of *Alpha*- and *Betaproteobacteria*. In freshwater ecosystems, *Actinobacteria* are very abundant, while *Gammaproteobacteria* represent a tiny minority when there are no cyanobacterial blooms (e.g., Humbert et al., 2009). In addition, the abundance of these *Actinobacteria* decreases while the abundance of *Gammaproteobacteria* increases during cyanobacterial blooms (Parveen et al., 2013; Ghai et al., 2014), which we observed in both cultivated and natural BCs. At the OTU level, there were large differences between cultivated and natural communities, but a small number of OTUs were shared by natural and cultured BCs. These OTUs contained a high number of reads, indicating their presence among the core species of these BCs. BCs contain an assemblage of core and satellite species as defined by Gibson et al. (1999); the former are abundant and widely distributed, while the abundances of the latter are much more variable, and their distribution is limited to a restricted number of sites (Pedrós-Alió, 2006; Humbert et al., 2009). The presence of these core species in cultivated BCs supports their ability to survive under culture conditions and to play an important role in community functions.

Finally, when considering our data in terms of the potential utility of these cultivated communities to study the interactions between heterotrophic bacteria and cyanobacteria, the complexity of the cultivated communities and the presence of OTUs belonging to the core species of natural communities suggest that these cultivated strains are good models for further studies.

The second aim of this study was to compare the BCs associated with cyanobacterial strains belonging to four genera. Interestingly, these BCs displayed differences in their structural and functional diversity. In particular, BCs associated with N_2_+ genera cultivated in Z8X medium (*Aphanizomenon* and *Dolichospermum*) were clearly distinguishable from those associated with N_2_- genera cultivated in Z8 medium (*Microcystis* and *Planktothrix*). Within these two groups, separation between BCs according to genus was less evident. Significant congruence of the results obtained from the EcoPlate and pyrosequencing approaches was noted; thus, differences in the OTU composition of BCs associated with cyanobacteria in cultures likely correspond to differences in their ability to use different carbon sources.

One of the major questions raised by these findings concerns the relative importance of the cyanobacteria versus that of the culture media in terms of the differences observed in BCs. In natural aquatic ecosystems (Sarmento and Gasol, 2012; Landa et al., 2014) and in culture (Bagatini et al., 2014; Landa et al., 2014), the composition of microbial communities is primarily influenced by the quality and quantity of OM available in these systems. Furthermore, N_2_+ cyanobacteria release significant amounts of NH_4_^+^ (Mulholland, 2007; Ploug et al., 2011; Adam et al., 2016), which is used by the surrounding BC and consequently prevents putative BC limitation by nitrogen in Z8X media. In this study, the EcoPlate approach revealed a greater NUI for BCs associated with N_2_- cyanobacteria cultivated in Z8 medium than for BCs associated with N_2_+ cyanobacteria cultivated in Z8X medium without nitrogen; this difference may be a consequence of potential nitrogen limitation in BCs associated with N_2_- cyanobacteria. However, Brauer et al. (2015) recently demonstrated competition and facilitation between a marine N_2_-fixing cyanobacterium and its associated BC and found the influence of nitrogen and carbon availability on the dynamics and composition of BCs associated with the cyanobacterium to be highly complex and dependent on carbon availability and physical parameters such as temperature. It is very difficult to evaluate the relative importance of the culture medium and of nitrogen fixation by cyanobacteria on the observed differences between BCs associated with N_2_+ and N_2_- cyanobacteria; thus, new studies focusing specifically on the nitrogen cycle will facilitate a better answer to this question.

The second interesting finding obtained by comparing the structure and composition of BCs associated with cyanobacteria in culture was the observation of two major categories of dominant OTUs in these BCs: OTUs specifically associated with cyanobacteria or other phytoplankton species (phyto-OM specialized bacteria) and OTUs found in environments characterized by high quantities of OM, such as sludge, human waste, and the rhizosphere (generalist-OM bacteria). Interestingly, several OTUs that were widely distributed among both our cultivated BCs and in natural environments are known to potentially fix N_2_. The majority of these were closely related (i) to the genus *Rhizobium*, known to engage in symbiotic relationships with plant roots and to fix free nitrogen, even when free-living (Dreyfus et al., 1983; Ludwig, 1984; Alazard, 1990); (ii) to the genus *Azospirillum*, known to contain endophytic diazotrophic rhizobacteria (Santi et al., 2013); and (iii) to the genus *Pseudomonas*, which contains certain species that were recently described as potential N_2_ fixers in marine environments (Dang et al., 2013). Further studies on the diversity and the expression of functional genes such as *nifH* will permit to estimate the contribution of these potentially diazotrophic bacteria in the nitrogen cycle during cyanobacterial cultures. For example, in marine environments it has been shown that some Gammaproteobacterial diazotrophs could have a significant contribution to N budget in these ecosystems (Moisander et al., 2014). Moreover, it would be interesting to distinguish BCs that are attached to cyanobacteria and organic aggregates, from the free living BCs because it has been shown that suboxic conditions in aggregates may promote the N_2_ fixation activity of heterotrophic diazotrophs (see review of Dang and Lovell, 2016).

## Conclusion

Our study revealed the complex structures of BCs associated with cultivated cyanobacterial strains, which contained several abundant OTUs that were also highly abundant in BCs associated with natural cyanobacterial blooms. These results highlighted the relevance of the culture models employed to study interactions between bacteria and cyanobacteria. An additional, original finding of this study is the observation that BCs associated with different cyanobacterial genera exhibited functional and structural differences related to the phylogenetic relationships among these cyanobacterial genera and, potentially, to the quality of the OM produced. Finally, BCs associated with cyanobacteria were composed of a variety of OM-generalist and specialist species, which will be explored in our future work.

## Author Contributions

LZ, AZ, IL, SD, CB, LS, JL, and J-FH have worked on the design of the experiments. LZ, AZ, IL, SD, CD, and KT have performed the experiments. LZ, AZ, IL, DD, JL, and J-FH have performed the data analysis. CD and KT help to the data analysis. LZ, AZ, IL, CD, CB, DD, LS, JL, and J-FH have written the paper. J-FH co-led the project with JL, CB, and LS.

## Conflict of Interest Statement

The authors declare that the research was conducted in the absence of any commercial or financial relationships that could be construed as a potential conflict of interest.

## Footnotes

1 http://github.com/panammeb/

## Supplementary Material

FIGURE S1 **Principal component analyses performed on the OTU composition (pyrosequencing approach) of the BCs associated with the two natural blooms of *Microcystis* and *Dolichospermum* and the 15 cyanobacterial strains in culture.** For each strain, the replicates are connected to facilitate reading. Env. *Dolichospermum* and Env. *Microcystis*: environmental samples from a *Dolichospermum* bloom and a *Microcystis* bloom, respectively.

TABLE S1 **Description of the results obtained from the BLAST analysis performed on the 100 most abundant OTUs found in BCs associated with cyanobacteria in culture**.
