# Supplementary figures and images for: Bacterial Communities Associated with Four Cyanobacterial Genera Display Structural and Functional Differences: Evidence from an Experimental Approach
Source: Front Microbiol. 2016 Oct 24;7:1662. doi: 10.3389/fmicb.2016.01662 (PMC5076464; doi:10.3389/fmicb.2016.01662)

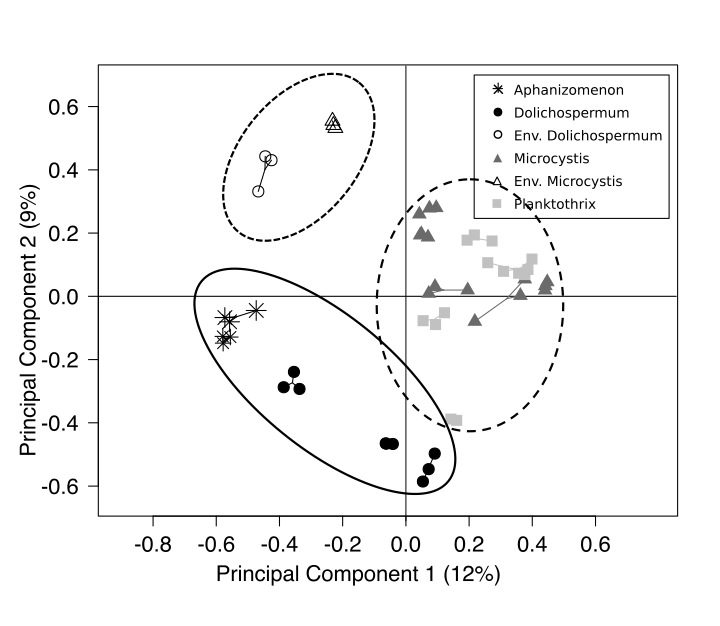

Supplement: FIGURE S1 — Principal component analyses performed on the OTU composition (pyrosequencing approach) of the BCs associated with the two natural blooms of Microcystis and Dolichospermum and the 15 cyanobacterial strains in culture. For each strain, the replicates are connected to facilitate reading. Env. Dolichospermum and Env. Microcystis: environmental samples from a Dolichospermum bloom and a Microcystis bloom, respectively. [file Image_1.JPEG]
